# Kernel Principal Component Analysis of Coil Compression in Parallel Imaging

**DOI:** 10.1155/2018/4254189

**Published:** 2018-04-19

**Authors:** Yuchou Chang, Haifeng Wang

**Affiliations:** ^1^Computer Science and Engineering Technology Department, University of Houston-Downtown, Houston, TX 77002, USA; ^2^Shenzhen Institutes of Advanced Technology, Chinese Academy of Sciences, Shenzhen, Guangdong 518055, China

## Abstract

A phased array with many coil elements has been widely used in parallel MRI for imaging acceleration. On the other hand, it results in increased memory usage and large computational costs for reconstructing the missing data from such a large number of channels. A number of techniques have been developed to linearly combine physical channels to produce fewer compressed virtual channels for reconstruction. A new channel compression technique via kernel principal component analysis (KPCA) is proposed. The proposed KPCA method uses a nonlinear combination of all physical channels to produce a set of compressed virtual channels. This method not only reduces the computational time but also improves the reconstruction quality of all channels when used. Taking the traditional GRAPPA algorithm as an example, it is shown that the proposed KPCA method can achieve better quality than both PCA and all channels, and at the same time the calculation time is almost the same as the existing PCA method.

## 1. Introduction

Parallel imaging methods [1, 2] have been widely used to accelerate MRI acquisitions. Due to the increased number of coils in parallel Magnetic Resonance Imaging, the numbers of coils (e.g., 128 channels) have been developed to improve the image quality of reconstruction and the sampling speed of acquisition [3–5]. On the other hand, the calculated cost increases as the number of coils increases, especially for coil-based reconstruction methods such as GRAPPA [2]. A number of coil compression methods have been proposed [6–19] to reduce computational time. They can be divided into two categories, one based on the hardware approach [5] and the other based on the software approach [6–19]. Those software-based coil compression methods provide a more flexible way to reduce computation workload. For example, principal component analysis (PCA) has been applied on compressing large array coils [10, 19]. The coil compression process produces a smaller set of virtual channels that can be represented as a linear combination of physical channels. The method has been successfully applied to most existing reconstruction methods such as SENSE [1], GRAPPA [2], and SPIRiT [20]. All existing coil compression methods have demonstrated that the number of channels can be greatly reduced without significant loss of SNR or image degradation, thereby increasing computational efficiency. In addition to saving computing time, PCA-based channel reduction methods have been shown to have noise reduction effects [10, 19, 21]. However, this denoising effect has been discussed in [21] without significant improvement.

The purposed method is to study the noise reduction capability of software-based coil compression methods while achieving noise suppression and channel reduction simultaneously. And we present a PCA-based approach, which is a nonlinear extension of the conventional PCA method [10, 19]. In contrast to the linear combination used in the conventional PCA, the proposed channel reduction technique nonlinearly combines physical channels to generate a new reduced set of virtual channels. Actually, the conception of nonlinear reconstruction using kernel methods has been studied in nonlinear GRAPPA [22], and the advantages of nonlinear combination over linear techniques have been demonstrated. The proposed kernel PCA (KPCA) method can reduce the usage of nonlinear combination on additional dimensions and more effectively enhance the quality of coil channels. In experiments, we used the GRAPPA method [2] as the reconstruction demos to achieve the final images from the data reduced channels. When generating the same small number of virtual channels, the proposed KPCA can reduce GRAPPA calculation time the same as the previous PCA-GRAPPA reconstruction [10] calculation time; however, the signal-to-noise ratio (SNR) is higher than the conventional GRAPPA [2] and PCA-GRAPPA [10].

## 2. Background

Generally, the GRAPPA reconstruction [2] can be represented as

(1) Sjky+rΔky,kx=∑l=1L ∑b=B1B2 ∑h=H1H2wj,rl,b,h×Slky+bRΔky,kx+hΔkx,

where the unacquired *k*-space signal *S*_*j*_ (the left side of (1)) is calculated by a linear combination of *k*-space signals (the right side of (1)). Here, *w* represents the coefficient set, *R* is the outer reduction factor (ORF), *j* is the target coil, *l* counts all coils, *b* and *h* are calculated by taking neighbored *k*-space data in *k*_*y*_ and *k*_*x*_ directions, respectively, and the variables *k*_*x*_ and *k*_*y*_ represent the coordinates encoded along the frequency and phase, respectively. The GRAPPA formulation can be simplified as a matrix equation:

(2) $$\begin{array}{c} b_{M\times 1}=D_{M\times K}x_{K\times 1}, \end{array}$$

where** D **denotes the matrix consisting of the acquired data,** b** represents the vector of the missing data, and** x** represents the coefficients.

In general, the coefficients are dependent on the coil sensitivity, which are a priori unknown. In GRAPPA, autocalibration data (ACS) are obtained and used as the vector** b** to estimate the coefficient vector** x**. The least-squares method is usually used to calculate the coefficients:

(3) $$\begin{array}{c} \hat{x}=\underset{x}{arg\;\;min}\;{\left\|\;b-Dx\;\right\|}^{2}. \end{array}$$

When the matrix** D** changes with a higher reduction factors, the noise in the estimation coefficients can be greatly amplified.

As a dimension reduction technique, PCA has been successfully used to reduce the number of effective channels in GRAPPA reconstruction [8, 9]. The PCA finds an orthogonal linear transformation that converts the data to a new coordinate system so that the largest change in any projection of data comes from the first coordinate, the second largest change is on the second coordinate, and so on. When applied to channel reduction, the ACS data is used to obtain the transformation and then applied to all acquired data to obtain a new dataset in the new coordinate system. Mathematically, the linear transformation** W** can be calculated by the eigen-decomposition of the covariance matrix of the ACS data:

(4) $$\begin{array}{c} A^{H}A=W^{H}\Sigma W, \end{array}$$

where **A** = [**a**_1_, **a**_2_,…, **a**_*L*_] consisted of vector **a**_*l*_ generated from the ACS data of the *l*th channel (a total of* L* channels) after removing the average;** W** and Σ are, respectively, eigenvectors and eigenvalues of the matrices. The new coordinates based on eigenvectors are called principal components. Assuming that the direction of largest variance represents interesting information and the direction of the minimum variance indicates noise that is not of interest. For simplicity, only a few first eigenvectors corresponding to the largest eigenvalues are retained to form a linear transformation** T**. The transformation matrix is then applied to the acquired *k*-space data to obtain an orthogonal projection of the eigenvectors, resulting in a new set of reduced virtual channels. Then, the undersampled data is reconstructed in the transform domain by conventional GRAPPA. Note that [9] the number of source channels (*N*_sch_) and the number of target channels (*N*_tch_) may differ after PCA reduction. One may be bigger than the other, with the same calculation time to get the best result. The final image is produced by combining the virtual channels with root sum-of-square (SOS). Obviously, the assumptions in the PCA are not necessarily kept because of the possibility of small variance in the direction of interesting signals, in which case the useful information is lost after reductions.

## 3. Proposed Method

### 3.1. Kernel PCA

The kernel method [23] is a widely used machine learning method. The main idea of the kernel method is that a set of points which cannot be linearly segmented in a low-dimensional space is likely to become linearly separable when transformed into a set of points in a high-dimensional space. For a given linear algorithm, the data is mapped from the input space *A* to the feature space* H *through a nonlinear mapping Φ(·): *A* → *H*, and then the algorithm is applied on the vector representation Φ(**a**) of the data. When the PCA method is a nonlinear mapping algorithm, the approach becomes a kernel PCA (KPCA) method.

PCA is a process of attribute dependency. The correlation here mainly refers to the linear correlation. So, for nonlinear situation, it involves kernel PCA called KPCA [24]. Intuitively, the kernel PCA is the PCA dimensionality reduction based on the kernel space after the original sample has passed the kernel mapping. KPCA formula derivation and PCA are very similar, but there are two innovations. In order to deal with nonlinear data better, a nonlinear mapping function Φ(**A**) is introduced to map the data in the original space into a high-dimensional feature space. For any vector in space, even if it is a basis vector, all samples lie in the linear representation. After kernel mapping, we make a linear PCA on the new data in the feature space constructed by the product of vector elements, thus taking into account higher-order statistics. We applied kernel PCA on parallel imaging reconstruction methods such as GRAPPA [2].

### 3.2. Nonlinear Mapping Function

In order to achieve a smooth relationship, a nonuniform polynomial kernel is selected for Φ mapping. It has the following form:

(5) $$\begin{array}{c} \kappa \left(\;a,b\;\right)={\left(\;\lambda a^{T}b+r\;\right)}^{d}, \end{array}$$

where *λ* and *r* are scalars; *d* represents the degree of the polynomial. Due to explicit representation of nonlinear mapping Φ(**A**) of *κ*(**a**, **b**) = 〈Φ(**a**), Φ(**b**)〉, polynomial kernel is also suitable for mapping MRI data. For instance, Φ(**A**) maps the original* L*-channel data** A** to **a**^2^, when *d* is 2,

(6) ФA=r2,2λra1,…,2λraL,λa12,…,λaL2,2λa1×a2,…,2λai×aj,…,2λaL−1×aLT,

where **a**_1_, **a**_2_,…, **a**_*L*_ are vectors representing different channels; superscript (2) means piecewise square; × denotes piecewise multiplication. It can be seen that the vector includes the constant, linear, second-orders in the original data, and Φ(**A**) has (*L* + 2)(*L* + 1)/2 terms in total.

In order to avoid overfitting, some second-order terms are removed. In particular, the second-order terms are rearranged in the following order. The square terms are selected within each coil at first, and then the product terms between the nearest neighbors are chosen, and then the next-nearest neighbors are selected in *k*-space and so on. The vector Φ(**a**) is removed by using sorted terms based on the desired dimension of the feature space. If all second-order terms are truncated, the proposed method is the same as linear PCA-based channel compression algorithm.

The target channels are corresponding to data on the left side of (1) and source channels as those for the right side of (1). The original space for the target channel is selected to avoid the complexity of converting the data from the feature space back to the original space. The source channels are used for estimation only, so there is no need to convert it back to the original space. The number of second-order terms to be three times of that of the first-order terms is chosen for building the source channels. Since MRI noise is generated in a very complicated procedure, which can be considered as non-Gaussian distribution [25]. Noise also exists in sensitivities of acquired channel data. Noise and true signal can be considered as error-in-variable model [22]. The traditional linear space is mapped to nonlinear feature space to capture noise characteristics existing in coil sensitivities. Nonlinearity is added to modulate sensitivities in the channel compression procedure. The benefit of the proposed method is the simultaneous channel compression and noise suppression in reconstruction procedure.

To balance linearity and nonlinearity of the new coordinate system, the parameters *r* and *λ* are finely tuned. If the nonlinearity dominates the coordinate, the reconstructed image quality is distorted since the original channel information is lost and overridden by nonlinearity information. By contrast, if the nonlinearity is too tiny, reconstruction is almost equivalent to original PCA-based channel reduction method, so that nonlinearity does not have effect on suppressing noise. $\sqrt{2\lambda r}=1$ and adjustable *λ* are set to obtain the better performance. The maximum absolute value *M*_2nd_ of the second-order terms is identified for building the feature space. *λ*_*s*_ sets the value within the range within (1/*M*_2nd_, 10/*M*_2nd_) based on the experience that the reconstruction is insensitive to the values in the above range.

### 3.3. Proposed Algorithm

The proposed method is presented in the following steps.

**Step 1 .**

Extracted calibration data is the input data of KPCA for target channels and source channels, respectively. The calibration data in each channel is arranged into a vector; therefore, there are overall *N* vectors **V**_1_, **V**_2_,…, **V**_**N**_ corresponding to overall *N* channels of original *k*-space data.

**Step 2 .**

Nonlinear mapping Φ is applied on random variable** V** here to construct the covariance matrix *C*_*t*_ and *C*_*s*_ of target channels and source channels, respectively. The new vectors** U** are constructed as follows:

(7) U=V1,V2,…,VN,λV11,λV22,…,λVNN,λVN1,λV12,λV23,…,λVN−1N,λVN−11,λVN2,λV13,λV24,…,λVN−2N,

where **V**_1_, **V**_2_,…, **V**_**N**_ denote vectors obtained from original *k*-space ACS data; second-order terms represent the vector from the point-wise multiplication by **V**_1_, **V**_2_,…, **V**_**N**_. For example, **V**_11_ = [*v*_1_^2^, *v*_2_^2^,…,*v*_*n*_^2^]^*T*^. Furthermore, the dimension of** U** is *n* × 4*N*, where *n* is the total number of *k*-space data obtained at the central strip, and* Nyquist* rate (*n* = *N*_nyq_ × *N*_*x*_, where *N*_nyq_ is the number of phase-encoding lines fully sampled with Nyquist rate, and *N*_*x*_ is the number of points along the frequency-encoding direction) is fully sampled. If ACS lines are defined, which are fully sampled *k*-space data at the central strip, *N*_acs_ = *N*_nyq_ can be derived. Nonlinearity controlled by the parameter *λ* is added into the new coordinates. Since the target channels will be used for final reconstruction which can't be incorporated large nonlinearity, so both parameters *λ*_*t*_ and *λ*_*s*_ are tuned for constructing** U**_**t**_ and** U**_**s**_, respectively. Generally, *λ*_*t*_ is much smaller than *λ*_*s*_.

**Step 3 .**

For target and source vectors** U**_**t**_ and** U**_**s**_ produced in Step 2, mean of zero is calculated to make sure** U**_**t**_ and** U**_**s**_ will be the direction of maximal variance. The mean of zero can be calculated as follows:

(8) $$\begin{array}{c} \hat{U}=U-\frac{\sum_{p=1}^{n}u^{p}}{n}. \end{array}$$

**Step 4 .**

For target channels, covariance matrix *C*_*t*_ is generated as follows:

(9) $$\begin{array}{c} C_{t}=cov\;\left(\;{\hat{U}}_{t}\left(\;i\;\right),{\hat{U}}_{t}\left(\;j\;\right)\;\right),\;1\leq i\leq 4N,\;1\leq j\leq 4N, \end{array}$$

where one component *C*_*t*_(*i*, *j*) represents covariance between random variables ${\hat{U}}_{t}\left(i\right)$ and ${\hat{U}}_{t}\left(j\right)$. The parameter *λ*_*t*_ can be set as zero to keep uniform with PCA-based channel reduction.

**Step 5 .**

Similarly to Step 4, covariance matrix *C*_*s*_ is constructed for source channels. The difference is that the parameter *λ*_*s*_ is chosen, which is generally larger than *λ*_*t*_ in Step 4.

**Step 6 .**

Calculate eigenvalues and eigenvectors using singular value decomposition (SVD) on covariance matrix *C*_*t*_ and *C*_*s*_, respectively. Since nonlinear mapping is directly used here and kernel trick matrix is not needed to be computed here, SVD can be directly used here to calculate eigenvalues and eigenvectors, like conventional linear PCA does [23]. The transformation matrix** T** is composed of eigenvectors of the covariance matrix, which transforms data in** U**_**t**_ and** U**_**s**_ into new coordinates.

**Step 7 .**

Generate the transformed data in the new coordinates for *N*_tch_ target channels and *N*_sch_ source channels, respectively. Similarly to [26], *N*_tch_ and *N*_sch_ are not needed to be necessarily equal. In the calibration step, *S*_*l*_ in (1) are obtained from target channels and *S*_*j*_ in (1) are obtained from source channels to calculate weights. In the synthesis step, calculated weights and acquired data on source channels are combined to predict missing values on target channels, which are used for final image reconstruction.

## 4. Results

We validate the proposed algorithm performance by using three MRI datasets. At first, a uniform water phantom was scanned using a gradient echo (GRE) sequence (15.63 kHz bandwidth, FOV = 250 mm^2^, matrix size = 256 × 256, TE/TR = 10/100 ms, and slice thickness = 3.0 mm). Then, a coronary brain image was acquired by using a 2D spin echo (SE) sequence (slice thickness = 3.0 mm, matrix size = 256 × 256, FOV = 240 mm^2^, and TE/TR = 2.29/100 ms,). The third set of axial brain data was acquired on a 3T scanner (SIEMENS AG, Erlangen, German) with a 32-channel head coil using a 2D gradient echo sequence (TE/TR = 2.29/100 ms, flip angle = 25, matrix size = 256 × 256, slice thickness = 3 mm, and FOV = 24 cm^2^). The conventional GRAPPA [2] and PCA-based GRAPPA [10] were implemented for comparing with the proposed method on the Matlab platform (Mathworks, Natick, MA, USA). For reference image, fully sampled data with all channels are reconstructed via root sum of squares (SoS).

**Figure 1:**
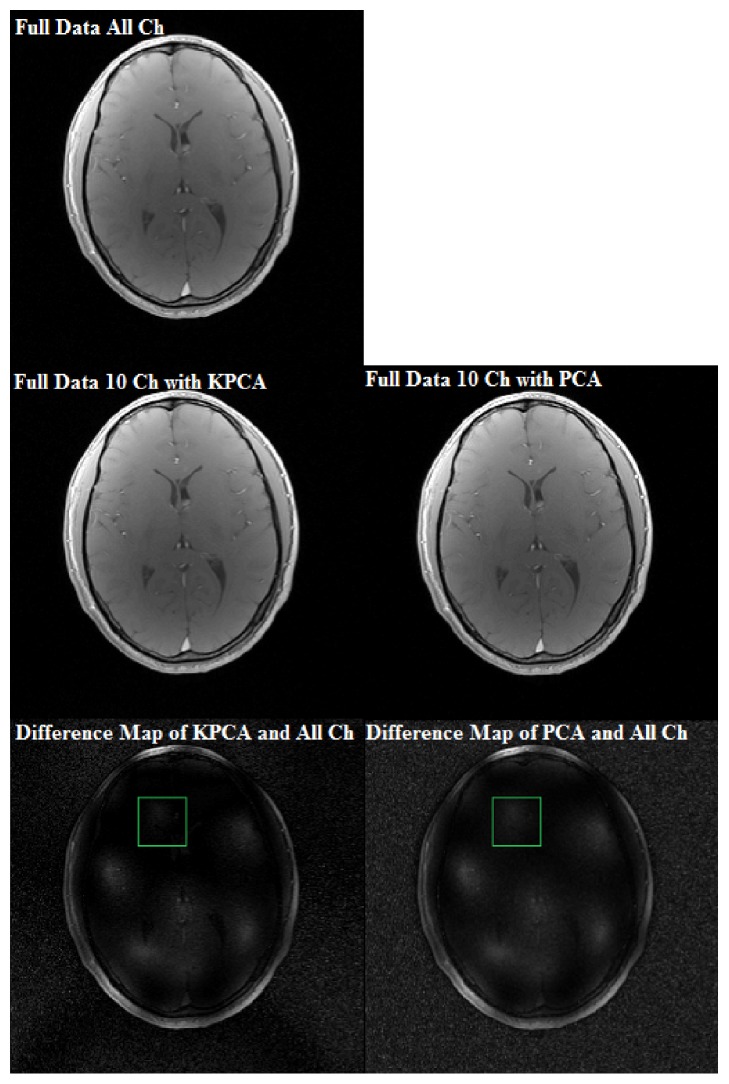
Reconstruction comparison of PCA and KPCA with compressed data. All channel (All Ch) data are used for the reference image. And the reconstructed images of KPCA and PCA with 10-channel (10 Ch) compressed data are, respectively, compared with the reference image.

To measure signal loss in channel compression, KPCA and PCA channel reduction based reconstructions with fully sampled data are evaluated firstly. Both of KPCA and PCA are applied to reduce the total 32 channels to 10 channels without undersampling *k*-space data. The compressed channels are used to reconstruct the images with inverse Fourier transform, respectively. Both reconstructed images are compared to the reference image with fully sampled data of all 32 channels. As shown in Figure 1, KPCA channel compression based reconstruction can suppress more noise than PCA channel compression based reconstruction in the region of interest (ROI), as demonstrated in the difference images.

**Figure 2:**
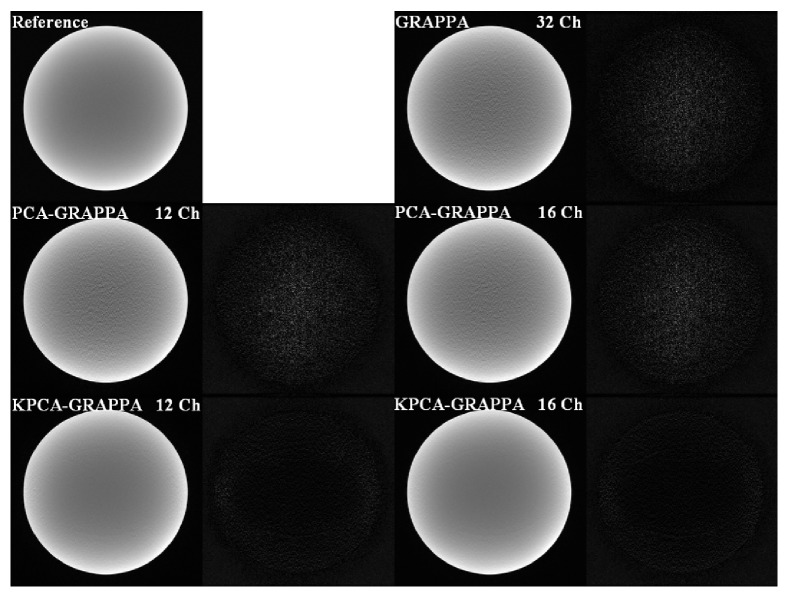
Reconstruction results of the 32-channel (32 Ch) experimental phantom images. Three methods were compared, the GRAPPA using full channel data, the PCA-GRAPPA, and KPCA-GRAPPA using, respectively, 12-channel (12 Ch) and 16-channel (16 Ch) compressed data.

Figure 2 shows the reconstructions of the first dataset (phantom) reconstructed by the conventional GRAPPA with full channels, GRAPPA with PCA-based channel reduction, and the proposed method using KPCA-based channel reduction. The first dataset was undersampled with an outer reduction factor (ORF) of 5 and the ACS of 42 (net acceleration of 3.01). The configuration of the reconstruction coefficients was 15 columns and 2 blocks. The number of target channels is set as 12 and the number of source channels is set as 16 for comparing performance. All sources of errors are displayed by subtracting reconstructed image by reference images. The error resources include blurring, aliasing, and noise. The proposed method is able to suppress more noise in comparison with traditional GRAPPA with full channels and GRAPPA with PCA-based channel reduction.

**Figure 3:**
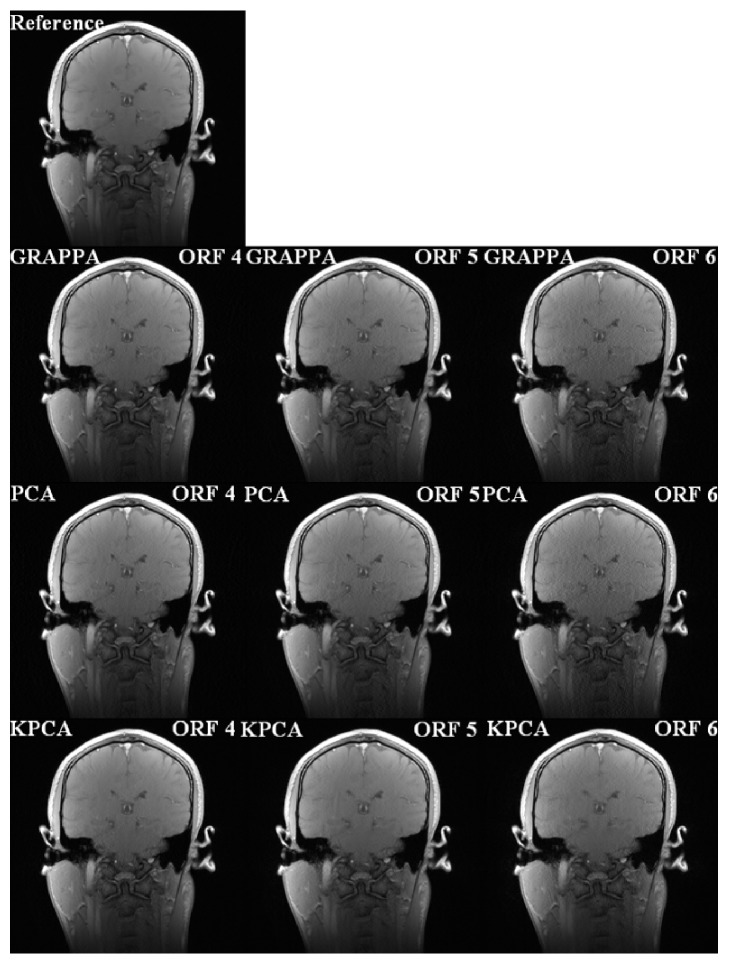
Reconstruction comparisons of the full 32-channel coronal human brain images. Three methods, the GRAPPA using full 32-channel data, the PCA-GRAPPA, and KPCA-GRAPPA using 20-channel compressed data, were compared, respectively, at the outer reduction factors (ORF) of 4, 5, and 6.

For the second dataset, reconstruction parameters are 48 ACS lines, 15 columns, and 2 blocks. Outer reduction factor (ORF) is set as 4, 5, and 6 for multiple comparisons. Correspondingly, the net reduction factors are, respectively, 2.56, 2.81, and 3.01. For PCA-GRAPPA and KPCA-GRAPPA, the number of reduced channels is set as 20 for both *N*_tch_ target channels and *N*_sch_ source channels. The parameter *λ*_sch_ is set as 1.16 × 10^−6^. From left column to right column, it is seen that reconstructions of GRAPPA, PCA-GRAPPA, and KPCA-GRAPPA are deteriorated when ORF is increasing. For the same ORF (from top to bottom), reconstruction of the proposed method always suppress more noise than that of traditional GRAPPA and GRAPPA with PCA-based channel reduction. For the column images of ORF 5, although trivial aliasing artifacts exist in all reconstructions of GRAPPA, PCA-GRAPPA, and KPCA-GRAPPA, the proposed method can reconstruct the image with cleaner contents as shown in Figure 3. Due to loss of information, reconstruction of PCA-GRAPPA is a very little worse than GRAPPA.

**Figure 4:**
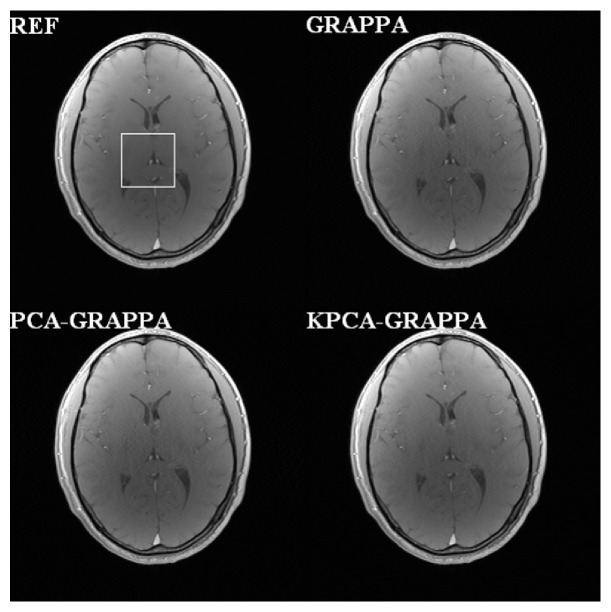
Reconstruction results of the 32-channel axial human brain images. We compared the GRAPPA reconstructions without channel reduction, with the PCA and KPCA-based channel reductions, when ORF = 4 and ACS = 48.

**Figure 5:**
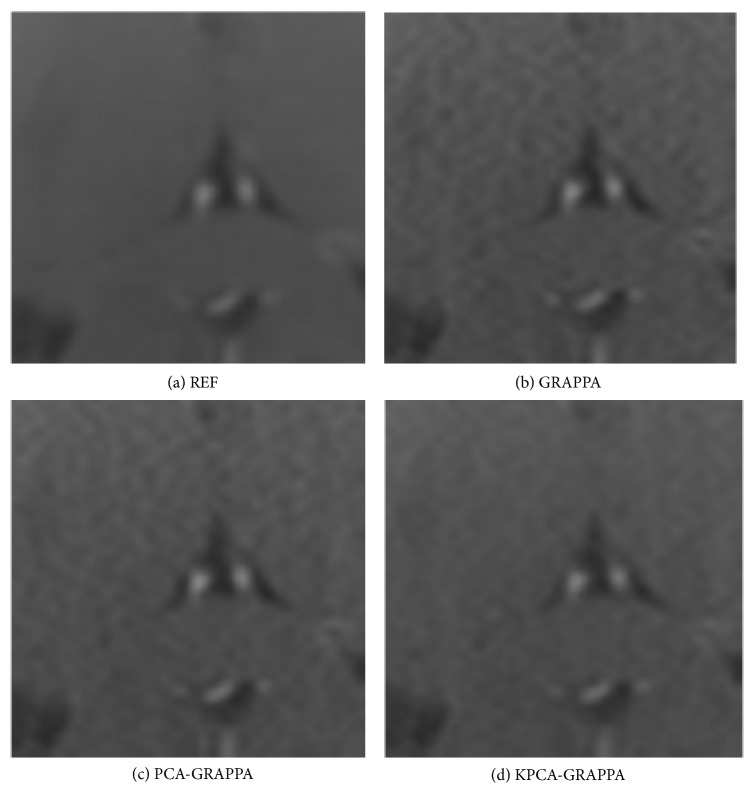
Zoom-in regions (white color box) of the reconstruction images in Figure 4. The proposed KPCA-GRAPPA method has higher SNR performance than GRAPPA and PCA-GRAPPA.

The proposed method is also evaluated and compared with the PCA-based channel reduction method shown in Figure 4 when using the traditional GRAPPA as the reconstruction method of the third dataset. It is also shown that there is no channel reduction in GRAPPA reconstruction and complete sampling from all channels as the square root of the reference image reconstructed for comparison. For visual comparison, the corners of each image contain an enlarged area of interest. Using 4 ORF and 48 ACS, the net acceleration was 2.56. The number of target and sources channels is 16, and *λ*_*s*_ is 1.23 × 10^−9^. Spatially varying noise is suppressed in both conventional GRAPPA and PCA-reduced GRAPPA reconstructions. Furthermore, the proposed method also preserves the image details without obvious blurring. The computation speed of the proposed method is almost the same as the PCA-based channel reduction method (around 863 seconds), which takes only about 11% reconstruction time of the traditional GRAPPA (7771 seconds) to reconstruct the image but the quality is better. Selecting a small area of interest to calculate the SNR of each reconstruction is shown in Figure 5. The SNRs of reference, conventional GRAPPA, PCA, and KPCA reduced GRAPPA reconstructions are 16.69 dB, 14.86 dB, 14.47 dB, and 15.42 dB, respectively.

## 5. Conclusion

A new nonlinear kernel PCA-based channel compression method is proposed in parallel MRI. The method maps data to higher dimensional space by nonlinear transformation and performs PCA to generate a set of compressed coils. The experimental results show the proposed coil compression method based on KPCA can not only reduce the computation time cost but also suppress more noise in GRAPPA reconstructions than previous PCA method. In the future, we plan to do more experiments and investigate how to automatically choose the optimal nonlinear mapping parameters of the proposed algorithm for more parallel imaging reconstructions [27–29].
